# Supplementary material for: NAD+ controls circadian rhythmicity during cardiac aging
Source: Commun Biol. 2026 Mar 11;9:476. doi: 10.1038/s42003-026-09818-1 (PMC13039344; doi:10.1038/s42003-026-09818-1)
Supplement: Supplementary file 2 — Supplementary Information [file 42003_2026_9818_MOESM2_ESM.pdf]

Supplementary information

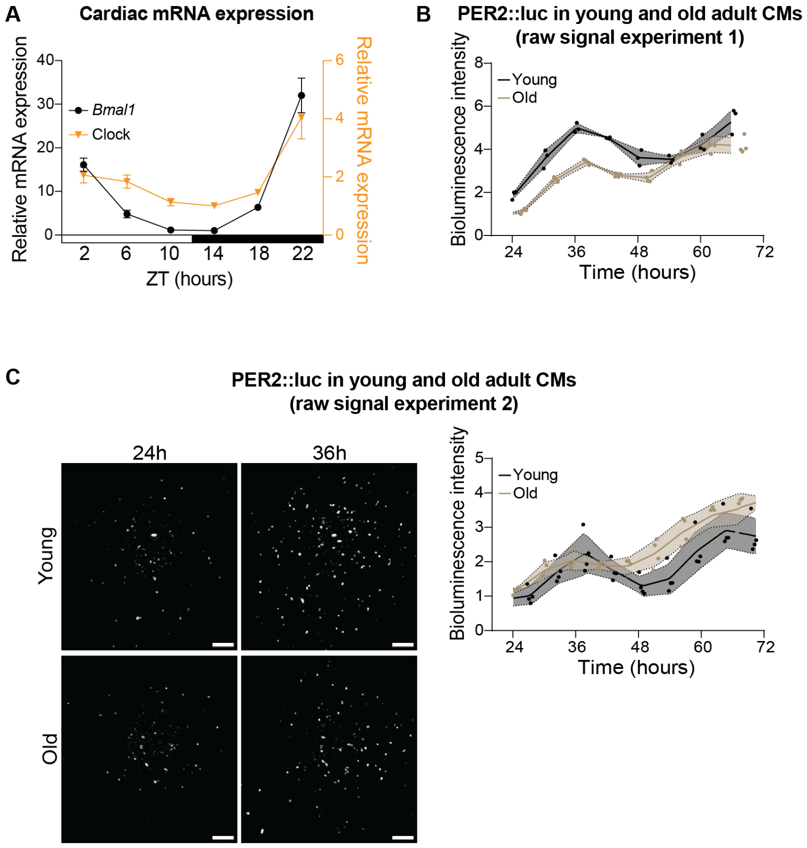

Supplemental Fig. 1.

(**A**) Circadian *Bmal1* and *Clock* mRNA expression in 12-week-old female mouse hearts (*n* = 4/timepoint). (**B**) Quantification of raw bioluminescence tracks from adult mouse cardiomyocytes isolated from young and old female Per2::luc animals in experiment 1 (*n* = 3 wells per age). **(C)** Representative bioluminescence captures and raw quantification of the second performance of the experiment in **(B)** (*n* = 3 wells per age). Circadian oscillations were analyzed using the COSINOR algorithm[1] to determine amplitude of rhythms by each well. Graphs show mean ± s.e.m.

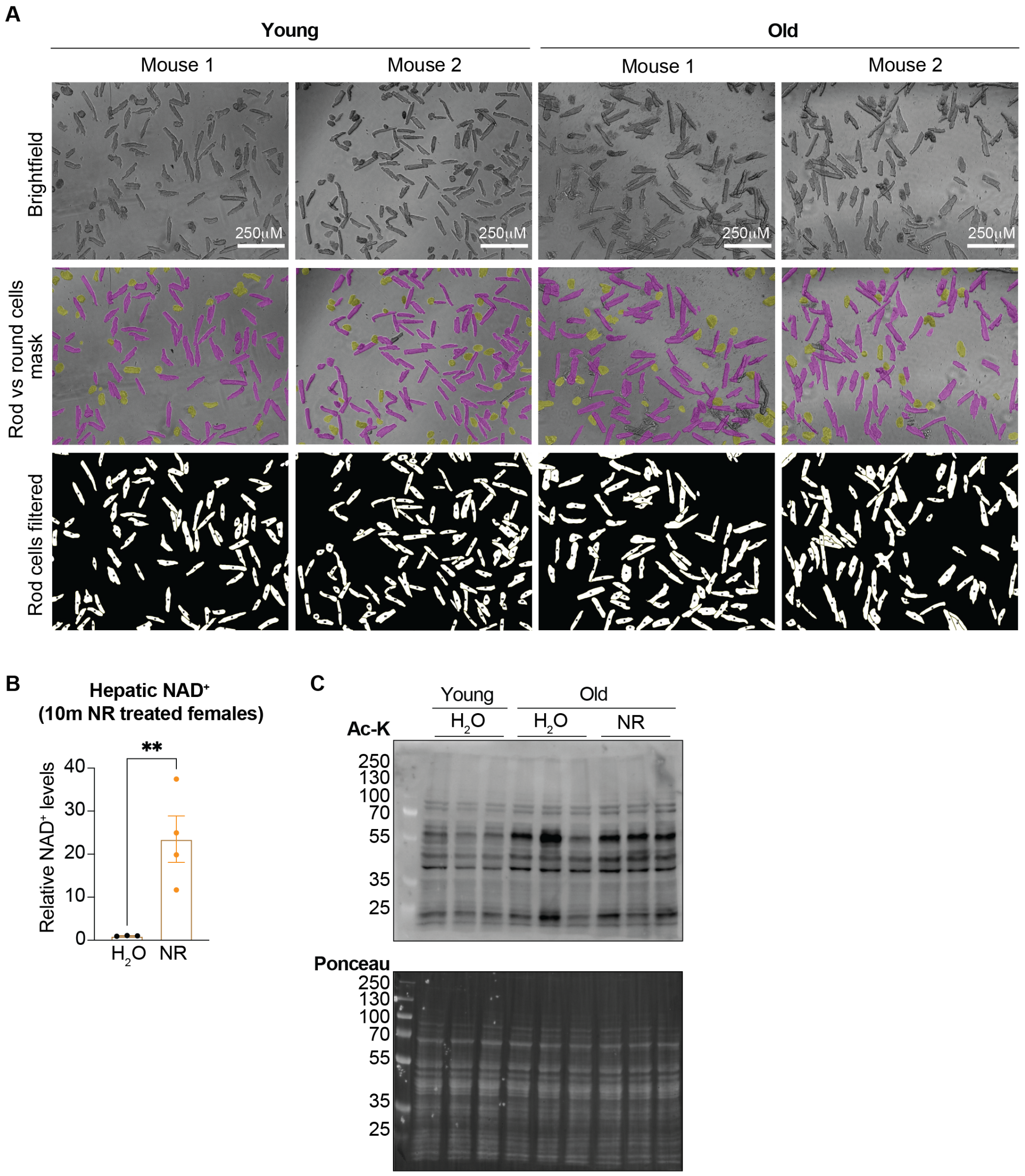

Supplemental Fig. 2.

(**A**) Workflow of analysis techniques and representative images used to capture the data of Figure 2C. Magenta indicates cells determined as rod-shaped by analysis macro, while yellow highlights rounded objects. Final mask was assessed manually to remove miscounts such as overlapping cardiomyocytes. (**B**) NAD^+ concentration in livers of female mice treated with NR versus H_2O (control) for 10 months starting at an age of 2 months at ZT10 (n = 3-4/treatment). (**C**) Western blots showing increased global acetylation of cardiac protein lysate on aging. **$p < 0.01$ by 1-tailed unpaired Student's t-test.

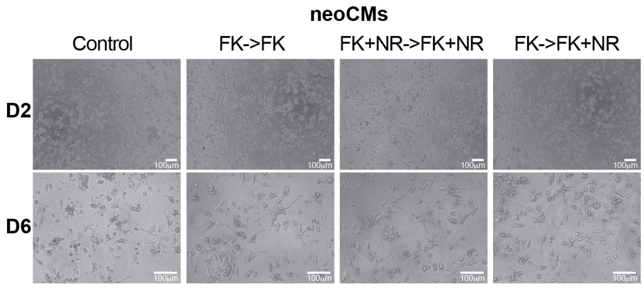

Supplemental Fig. 3.

Representative images of the neonatal mouse cardiomyocytes used in Figure 4A. Primary cells displayed typical signs of health such as robust and spontaneous contraction and beating. Treatments did not obviously impact cell density or activity. Scale bar represents 100μM.

A

Control vs FK866 (*n* = 841)

KEGG pathway	#Genes	*FDR*
ECM-receptor interaction	12	0.055
Protein digestion & absorption	13	0.055
MAPK signaling	25	0.066
Pathways in cancer	38	0.066
Amoebiasis	12	0.066
Breast cancer	15	0.066
Human papillomavirus infection	26	0.073
Focal adhesion	18	0.078
Nicotate and nicotinamide metabolism	6	0.066
Melanoma	9	0.091

FK866 vs FK866+NR (*n* = 618)

KEGG pathway	#Genes	*FDR*
Hepatocellular carcinoma	18	6.62×10^{-4}
Human papillomavirus infection	27	6.62×10^{-4}
Focal adhesion	19	0.0011
ECM-receptor interaction	12	0.0011
MAPK signaling pathway	23	0.0019
Breast cancer	15	0.0019
Pathways in cancer	34	0.0019
Gastric cancer	15	0.0019
Melanoma	10	0.0019
Proteoglycans in cancer	18	0.0019

FK866 vs FK866+NR+Ex527 (*n* = 213)

KEGG pathway	#Genes	*FDR*
ECM-receptor interaction	6	0.061
Transcriptional misregulation in cancer	8	0.092

SIRT1 dependent NR (*n* = 468)

KEGG pathway	#Genes	*FDR*
Human papillomavirus infection	21	0.0043
Hepatocellular carcinoma	14	0.0043
Gastric cancer	13	0.0043
Longevity regulating pathway	10	0.091
Breast cancer	12	0.094
Melanoma	8	0.096
Focal adhesion	14	0.096
Rheumatoid arthritis	9	0.096
Glioma	8	0.096
Axon guidance	13	0.096

SIRT1-independent NR (*n* = 150)

KEGG pathway	#Genes	*FDR*
ECM-receptor interaction	5	0.075

B

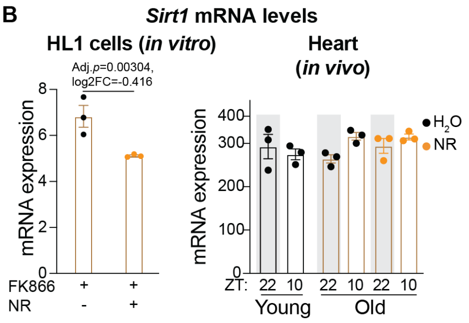

Supplemental Fig. 4.

(**A**) KEGG pathway analysis on the most prominent groups of differentially expressed genes described in Figure 5B/C. The analysis was performed via the use of Enrichr[2]. The top row corresponds to entire DEG lists for selected groups; the bottom row subdivides the “FK866 vs FK866+NR” group according to overlap or lack thereof with the “FK866 vs FK866+NR+Ex527” group. (**B**) mRNA expression counts of *Sirt1* in HL1 cells with labelled treatments (left, FPKM, Adj.*p* and $\log_2$FC by DESeq2) and young, old, and old NR-treated mice (right, DESeq2 normalized counts).

References

1. Molcan, L. Time distributed data analysis by Cosinor.Online application. *bioRxiv* 805960 (2023) doi:10.1101/805960.
2. Chen, E. Y. *et al.* Enrichr: interactive and collaborative HTML5 gene list enrichment analysis tool. *BMC Bioinform.* **14**, 128 (2013).
