# Supplementary material for: NAD+ controls circadian rhythmicity during cardiac aging
Source: Commun Biol. 2026 Mar 11;9:476. doi: 10.1038/s42003-026-09818-1 (PMC13039344; doi:10.1038/s42003-026-09818-1)
Supplement: Supplementary file 3 — Description of Additional Supplementary Files [file 42003_2026_9818_MOESM3_ESM.pdf]

Description of Additional Supplementary Files

File name: Supplementary Data 1
Description: Mouse adult heart RNA-seq processed data and KEGG analysis.

File name: Supplementary Data 2
Description HL-1 cell line RNA-seq processed data and KEGG analysis.

File name: Supplementary Data 3
Description: Source data for all figures, main and supplementary.
