# Supplementary material for: NAD+ controls circadian rhythmicity during cardiac aging
Source: Commun Biol. 2026 Mar 11;9:476. doi: 10.1038/s42003-026-09818-1 (PMC13039344; doi:10.1038/s42003-026-09818-1)
Supplement: Supplementary file 7 — Reporting Summary [file 42003_2026_9818_MOESM7_ESM.pdf]

nature portfolio

Corresponding author(s): Dierickx, Pieterjan

Last updated by author(s): Feb 6, 2026

Reporting Summary

Nature Portfolio wishes to improve the reproducibility of the work that we publish. This form provides structure for consistency and transparency in reporting. For further information on Nature Portfolio policies, see our Editorial Policies and the Editorial Policy Checklist.

Statistics

For all statistical analyses, confirm that the following items are present in the figure legend, table legend, main text, or Methods section.

n/a	Confirmed	
☐	☒	The exact sample size (*n*) for each experimental group/condition, given as a discrete number and unit of measurement
☐	☒	A statement on whether measurements were taken from distinct samples or whether the same sample was measured repeatedly
☐	☒	The statistical test(s) used AND whether they are one- or two-sided<br>*Only common tests should be described solely by name; describe more complex techniques in the Methods section.*
☐	☒	A description of all covariates tested
☐	☒	A description of any assumptions or corrections, such as tests of normality and adjustment for multiple comparisons
☐	☒	A full description of the statistical parameters including central tendency (e.g. means) or other basic estimates (e.g. regression coefficient) AND variation (e.g. standard deviation) or associated estimates of uncertainty (e.g. confidence intervals)
☐	☒	For null hypothesis testing, the test statistic (e.g. *F*, *t*, *r*) with confidence intervals, effect sizes, degrees of freedom and *P* value noted<br>*Give P values as exact values whenever suitable.*
☒	☐	For Bayesian analysis, information on the choice of priors and Markov chain Monte Carlo settings
☒	☐	For hierarchical and complex designs, identification of the appropriate level for tests and full reporting of outcomes
☒	☐	Estimates of effect sizes (e.g. Cohen's *d*, Pearson's *r*), indicating how they were calculated

Our web collection on statistics for biologists contains articles on many of the points above.

Software and code

Policy information about availability of computer code

Data collection	QuantStudio, cellSens, LumiCycle acquisition, NextSeq2000
Data analysis	QuantStudio, Prism10, R-Studios, Excel, Enrichr, COSINOR, CellPose, Ilastik, Trimmomatic version 0.39, STAR 2.7.10a, Picard 3.0.0, featureCounts 2.0.4, DESeq2 version 1.36.0, LumiCycle Analysis, Image J, Fiji

For manuscripts utilizing custom algorithms or software that are central to the research but not yet described in published literature, software must be made available to editors and reviewers. We strongly encourage code deposition in a community repository (e.g. GitHub). See the Nature Portfolio guidelines for submitting code & software for further information.

Data

Policy information about availability of data

All manuscripts must include a data availability statement. This statement should provide the following information, where applicable:

- Accession codes, unique identifiers, or web links for publicly available datasets
- A description of any restrictions on data availability
- For clinical datasets or third party data, please ensure that the statement adheres to our policy

The data that support the findings of this study are available in the main text and mRNA-seq data has been deposited in the NCBI Gene Expression Omnibus (GEO) under accession number GSE240483, linked to BioProject PPRJNA1002243.

Research involving human participants, their data, or biological material

Policy information about studies with human participants or human data. See also policy information about sex, gender (identity/presentation), and sexual orientation and race, ethnicity and racism.

Reporting on sex and gender	N/A
Reporting on race, ethnicity, or other socially relevant groupings	N/A
Population characteristics	N/A
Recruitment	N/A
Ethics oversight	N/A

Note that full information on the approval of the study protocol must also be provided in the manuscript.

Field-specific reporting

Please select the one below that is the best fit for your research. If you are not sure, read the appropriate sections before making your selection.

☒ Life sciences ☐ Behavioural & social sciences ☐ Ecological, evolutionary & environmental sciences

For a reference copy of the document with all sections, see nature.com/documents/nr-reporting-summary-flat.pdf

Life sciences study design

All studies must disclose on these points even when the disclosure is negative.

Sample size	Sample sizes were determined based on previous experimental observations and conventions derived from literature.
Data exclusions	One sample each was excluded from the day 2 and day 6 NADH measurements of Figure 4A in the FK866+NR groups; machine reported zero value on this specific well (technical error).
Replication	Data is typically displayed as one independent experiment. In cases of repeated experiments (Figure 1F and Supplementary Figure 1), all repetitions are reported separately and clearly stated as such in figure legends and text.
Randomization	Mice were selected for matching gender and age before the experiment, then randomly assigned to treatments.
Blinding	All experiments and analyses were performed blinded.

Reporting for specific materials, systems and methods

We require information from authors about some types of materials, experimental systems and methods used in many studies. Here, indicate whether each material, system or method listed is relevant to your study. If you are not sure if a list item applies to your research, read the appropriate section before selecting a response.

Materials & experimental systems

n/a	Involved in the study	
☐	☒	Antibodies
☐	☒	Eukaryotic cell lines
☒	☐	Palaeontology and archaeology
☐	☒	Animals and other organisms
☒	☐	Clinical data
☒	☐	Dual use research of concern
☒	☐	Plants

Methods

n/a	Involved in the study	
☒	☐	ChIP-seq
☒	☐	Flow cytometry
☒	☐	MRI-based neuroimaging

Antibodies

Antibodies used	anti-Acetyllysine (AKL5C1) : Santa Cruz Biotechnology, sc32268, https://datasheets.scbt.com/sc-32268.pdf

Validation	Validation according to the manufacturer's website (https://datasheets.scbt.com/sc-32268.pdf) is displayed with both experimental data and a reference list to use in literature.

Eukaryotic cell lines

Policy information about cell lines and Sex and Gender in Research

Cell line source(s)	The HL1 cell line was originally derived from atrial tumors of an adult C57BL6/J female mouse. The cells used in this experiments were obtained as a commercial product from Sigma Aldrich (SCC065) and maintained/expanded within the lab. Primary neonatal and adult cardiomyocytes were directly collected from PER2::Luc animals.
Authentication	All cells/protocols were previously authenticated by qPCR for marker gene expression. PER2::Luc animals were regenotyped prior to cell collection.
Mycoplasma contamination	All cells/cell lines were tested and reported negative for mycoplasma contamination.
Commonly misidentified lines (See ICLAC register)	*Name any commonly misidentified cell lines used in the study and provide a rationale for their use.*

Animals and other research organisms

Policy information about studies involving animals; ARRIVE guidelines recommended for reporting animal research, and Sex and Gender in Research

Laboratory animals	All animals used were species mus musculus, cross of C57BL6/J and C57BL6/N strains. Adult mice were all female, used at either 2-3 or 12-15 months of age. Neonates were used to collect cardiomyocytes 0-3 days after birth.
Wild animals	The study did not involve wild animals.
Reporting on sex	All experiments reported here were performed in female mice only. Female mice were purposefully selected prior to the experiment based on previous data that the response to the treatment strategy was greater than in male mice. All text and discussion clarifies that the conclusions are drawn from female mice alone.
Field-collected samples	The study not include samples obtained from the field.
Ethics oversight	All animal care and use procedures followed the guidelines of the Institutional Animal Care and Use Committee (IACUC) of the University of Pennsylvania and by the responsible Committee for Animal Rights Protection of the State of Hessen (Regierungspraesidium Darmstadt, Wilhelminenstr. 1-3, 64283 Darmstadt, Germany) with project number B2-2018.

Note that full information on the approval of the study protocol must also be provided in the manuscript.

Plants

Seed stocks	*Report on the source of all seed stocks or other plant material used. If applicable, state the seed stock centre and catalogue number. If plant specimens were collected from the field, describe the collection location, date and sampling procedures.*
Novel plant genotypes	*Describe the methods by which all novel plant genotypes were produced. This includes those generated by transgenic approaches, gene editing, chemical/radiation-based mutagenesis and hybridization. For transgenic lines, describe the transformation method, the number of independent lines analyzed and the generation upon which experiments were performed. For gene-edited lines, describe the editor used, the endogenous sequence targeted for editing, the targeting guide RNA sequence (if applicable) and how the editor was applied.*
Authentication	*Describe any authentication procedures for each seed stock used or novel genotype generated. Describe any experiments used to assess the effect of a mutation and, where applicable, how potential secondary effects (e.g. second site T-DNA insertions, mosiacism, off-target gene editing) were examined.*
